# Mortality from suicide among people living with HIV and the general Swiss population: 1988‐2017

**DOI:** 10.1002/jia2.25339

**Published:** 2019-08-18

**Authors:** Yann Ruffieux, Liis Lemsalu, Karoline Aebi‐Popp, Alexandra Calmy, Matthias Cavassini, Christoph A Fux, Huldrych F Günthard, Catia Marzolini, Alexandra Scherrer, Pietro Vernazza, Olivia Keiser, Matthias Egger, the Swiss HIV Cohort Study and the Swiss National Cohort, A Anagnostopoulos, M Battegay, E Bernasconi, J Böni, DL Braun, HC Bucher, A Ciuffi, G Dollenmaier, L Elzi, J Fehr, J Fellay, H Furrer, D Haerry, B Hasse, HH Hirsch, M Hoffmann, I Hösli, M Huber, CR Kahlert, L Kaiser, T Klimkait, RD Kouyos, H Kovari, B Ledergerber, G Martinetti, B Martinez de Tejada, KJ Metzner, N Müller, D Nicca, P Paioni, G Pantaleo, M Perreau, A Rauch, C Rudin, P Schmid, R Speck, M Stöckle, P Tarr, A Trkola, G Wandeler, R Weber, S TBD Yerly

**Affiliations:** ^1^ Institute of Social and Preventive Medicine (ISPM) University of Bern Bern Switzerland; ^2^ Department of Drug and Infectious Diseases Epidemiology National Institute for Health Development Tallinn Estonia; ^3^ Institute of Family Medicine and Public Health University of Tartu Tartu Estonia; ^4^ Division of Infectious Diseases Bern University Hospital University of Bern Bern Switzerland; ^5^ Division of Infectious Diseases University Hospital Geneva University of Geneva Geneva Switzerland; ^6^ Division of Infectious Diseases University Hospital Lausanne University of Lausanne Lausanne Switzerland; ^7^ Department of Infectious Diseases and Hospital Hygiene Kantonsspital Aarau Aarau Switzerland; ^8^ Division of Infectious Diseases and Hospital Epidemiology University Hospital Zurich Zurich Switzerland; ^9^ Institute of Medical Virology University of Zurich Zurich Switzerland; ^10^ Division of Infectious Diseases and Hospital Epidemiology Departments of Medicine and Clinical Research University Hospital Basel Basel Switzerland; ^11^ University of Basel Basel Switzerland; ^12^ Department of Infectious Diseases and Hospital Epidemiology Cantonal Hospital St Gallen St Gallen Switzerland; ^13^ Institute of Global Health University of Geneva Geneva Switzerland; ^14^ Centre of Infectious Disease Epidemiology and Research (CIDER) University of Cape Town Cape Town South Africa

**Keywords:** HIV, suicide, Switzerland, SMR, comparative study, intravenous drug use

## Abstract

**Introduction:**

In many countries, mortality due to suicide is higher among people living with HIV than in the general population. We aimed to analyse trends in suicide mortality before and after the introduction of triple combination antiretroviral therapy (cART), and to identify risk factors associated with death from suicide in Switzerland.

**Methods:**

We analysed data from the Swiss HIV Cohort Study from the pre‐cART (1988‐1995), earlier cART (1996‐2008) and later cART (2009‐2017) eras. We used multivariable Cox regression to assess risk factors for death due to suicide in the ART era and computed standardized mortality ratios (SMRs) to compare mortality rates due to suicide among persons living with HIV with the general population living in Switzerland, using data from the Swiss National Cohort.

**Results and Discussion:**

We included 20,136 persons living with HIV, of whom 204 (1.0%) died by suicide. In men, SMRs for suicide declined from 12.9 (95% CI 10.4‐16.0) in the pre‐cART era to 2.4 (95% CI 1.2‐5.1) in the earlier cART and 3.1 (95% CI 2.3‐4.3) in the later cART era. In women, the corresponding ratios declined from 14.2 (95% CI 7.9‐25.7) to 10.2 (3.8‐27.1) and to 3.3 (95% CI 1.5‐7.4). Factors associated with death due to suicide included gender (adjusted hazard ratio 0.58 (95% CI 0.38‐0.87) comparing women with men), nationality (1.95 (95% CI 1.34‐2.83) comparing Swiss with other), Centers for Disease Control and Prevention clinical stage (0.33 (95% CI 0.24‐0.46) comparing stage A with C), transmission group (2.64 (95% CI 1.71‐4.09) for injection drug use and 2.10 (95% CI 1.36‐3.24) for sex between men compared to other), and mental health (2.32 (95% CI 1.71‐3.14) for a history of psychiatric treatment vs. no history). There was no association with age.

**Conclusions:**

Suicide rates have decreased substantially among people living with HIV in the last three decades but have remained about three times higher than in the general population since the introduction of cART. Continued emphasis on suicide prevention among men and women living with HIV is important.

## 1 Introduction

Depression, anxiety and other mental health problems are common among people living with HIV, and more common than in the general population or among comparable HIV‐negative people 1, 2, 3, 4. In the era before the advent of triple combination antiretroviral therapy (cART), several studies showed that HIV infection was also associated with suicide 5, 6. In Switzerland, suicides accounted for 6% of deaths among people living with HIV 7. An analysis of the Swiss HIV Cohort Study (SHCS) found that during the years 1988 to 2008, suicide rates decreased somewhat with the introduction of cART, but remained above the rate observed in the general population. A recent study in the UK also found that in the cART era, HIV‐related suicide rates were higher than in the general population 8.

A French study reported that risk factors for suicide among persons living with HIV included a history of intravenous drug use (IDU), alcohol abuse and a history of depression or attempted suicide 9. In the Swiss study 10, a history of treatment by a psychiatrist was a predictor of suicide both before and after the introduction of cART, while factors such as advanced clinical stage and gender were important only before cART was introduced. Several antiretroviral drugs have been associated with adverse effects that could lead to suicide ideation 11, including efavirenz or dolutegravir, drugs which are widely used and recommended by the World Health Organization 12, 13, 14.

In this article, we update the previous analysis 10 of mortality rates due to suicide in persons living with HIV and the general population in Switzerland, to explore risk factors and time trends in suicide over almost 30 years, from the pre‐cART era up to recent years.

## 2 Methods

We analysed suicide rates and risk factors in the SHCS. Rates of suicide in people living with HIV were compared to suicide rates in the general Swiss population using data from the Swiss National Cohort (SNC). Throughout this study, the outcome of interest was death by suicide. No data were available on suicide ideation, or suicide attempts.

### 2.1 Data sources

The SHCS (www.shcs.ch) is an ongoing multicenter study involving people living with HIV aged 18 years or older 15. The study collects key patient information such as demographic characteristics, information on HIV‐related diseases and treatments, and laboratory measurements. This information is collected at enrollment and at six‐monthly follow‐up visits. The cohort is estimated to cover around 45% of the cumulative number of HIV infections notified to the Swiss health authorities, 69% of people living with AIDS and 75% of individuals receiving antiretroviral treatment 15.

The SNC (www.swissnationalcohort.ch) is a national longitudinal study on mortality, based on the probabilistic linkage of individual‐based data from 1990 and 2000 nationwide censuses with Federal mortality records 16. The cohort consists of 6.9 million census participants in 1990 and 7.3 million in 2000.

### 2.2 Data analysis

We compared characteristics of participants enrolled in the SHCS during the pre‐cART era (1988‐1995), earlier cART (1996‐2008), and later cART era (2009‐2017) between those who died by suicide (code ‘suicide’ in the SHCS, ICD‐8 codes E950‐959 and ICD‐10 codes X60‐X84 in the SNC) and those who did not. Completeness and quality of mortality data in the SHCS are high 7. For each year, we calculated the expected number of suicides, per sex and per five‐year age group, based on rates from the SNC data. From this we derived standardized mortality ratios (SMRs) for each ART era, assuming a Poisson distribution. We split the earlier cART era further into sub‐periods 1996‐1998 and 1999‐2008. We analysed predictors for suicide in the SHCS using multivariable Cox regression. This was done separately for the pre‐cART, earlier cART and later cART eras. We included the following predictors in the models: age at enrollment (per 10 years increase), gender, nationality (Swiss/other) and mode of transmission (injection drug use, men having sex with men, or other), Centers for Disease Control and Prevention (CDC) clinical stage as a time updated variable (A, B, or C), and prior treatment by a psychiatrist as a time‐updated variable. We used interaction tests to assess differences in associations between risk factors and suicide across time periods. We carried out a separate Cox regression analysis covering the period 2002 to 2017, which included socio‐economic factors: education (at enrollment, tertiary vs. other), living alone (time‐updated), and unemployment (time‐updated, defined as 50% or more of revenue provided by unemployment benefits since previous visit). We verified the Cox proportional hazards assumptions using Schoenfeld residual tests. In order to examine to what extent the trends observed were driven by specific patient groups, we recalculated SMRs after excluding individuals who had contracted HIV through IDU or through sex between men, and individuals treated by a psychiatrist at enrollment.

We identified predictors for efavirenz and dolutegravir use in patients on cART, using multivariate logistic regressions. Models were adjusted for age at enrollment, sex, nationality, transmission group, CDC clinical stage at enrollment, and history of psychiatric treatment at enrollment. Finally, we examined the association between efavirenz‐based therapy (time‐updated) and suicide among patients on cART, adjusting for the factors mentioned above. We performed a similar analysis for dolutegravir‐based therapy in the late cART era. Results are presented as adjusted hazard ratio (aHR) or adjusted odds ratio (aOR), with 95% confidence intervals.

## 3 Results and discussion

### 3.1 Characteristics of study population

We included 20,139 patients in the analysis. Participants enrolled in the later cART era were older and less likely to be women, Swiss nationals, or to have acquired HIV through injection drug use compared to those enrolled in the earlier cART era; they were also less likely to be in an advanced stage of HIV and more likely to be treated by a psychiatrist at enrollment (Table 1).

**Table 1:** Characteristics of participants in the Swiss HIV Cohort during the pre‐cART (1988‐1995), earlier cART (1996‐2008), and later cART (2009‐2017) eras, by whether or not they died by suicide

Variable	Pre‐cART era		Earlier cART era		Later cART era	
	Died by suicide		Died by suicide		Died by suicide	
	Yes (N = 92)	No (N = 7641)	Yes (N = 63)	No (N = 7793)	Yes (N = 49)	No (N = 4498)
Female	11 (12.0)	2040 (26.7)	14 (22.2)	2427 (31.1)	8 (16.3)	1052 (23.4)
Median age at enrollment (years)	31 (27‐39)	31 (27‐37)	34 (29‐44)	36 (30‐43)	36 (30‐43)	39 (31‐47)
Mode of transmission						
Intravenous drug use	42 (45.7)	3159 (41.3)	24 (38.1)	1357 (17.4)	14 (28.6)	228 (5.1)
Sex between men	41 (44.6)	2536 (33.2)	26 (41.3)	2806 (36.0)	25 (51.0)	2355 (52.4)
Heterosexual contact	9 (9.8)	1714 (22.4)	10 (15.9)	3282 (42.1)	9 (18.4)	1620 (36.0)
Other	0 (0.0)	232 (3.0)	3 (4.8)	348 (4.5)	1 (2.0)	641 (6.5)
CDC clinical stage						
At enrollment						
A	50 (54.3)	4421 (57.9)	40 (63.5)	5258 (67.5)	33 (67.4)	3433 (76.3)
B	24 (26.1)	1633 (21.4)	11 (17.5)	1258 (16.1)	6 (12.2)	448 (10.0)
C	18 (19.6)	1587 (20.8)	12 (19.1)	1277 (16.4)	10 (20.4)	617 (13.7)
At last visit						
A	25 (27.2)	1563 (20.5)	23 (36.5)	4123 (52.9)	21 (42.9)	3292 (73.2)
B	27 (29.3)	1843 (24.1)	18 (28.6)	1735 (22.3)	8 (16.3)	520 (11.6)
C	40 (43.5)	4235 (55.4)	22 (34.9)	1935 (24.8)	20 (40.8)	686 (15.2)
Psychiatric treatment						
At enrollment	10 (10.9)	400 (5.2)	12 (19.1)	599 (7.7)	4 (8.2)	457 (10.2)
Ever	19 (20.7)	2109 (27.6)	33 (52.4)	2819 (36.2)	28 (57.1)	1148 (25.5)
CD4 cell count (cells/μL)						
At enrollment						
Count available	80 (87.0)	6660 (87.2)	61 (96.8)	7780 (99.8)	48 (98.0)	4483 (99.7)
Median count	307 (125‐560)	306 (120‐540)	370 (195‐562)	339 (185‐526)	418 (194‐635)	420 (257‐608)
At last visit						
Count available	81 (88.0)	7154 (93.6)	62 (98.4)	7736 (99.3)	48 (98.0)	4448 (98.9)
Median count	180 (40‐440)	204 (30‐504)	286 (152‐532)	580 (382‐799)	477 (284‐712)	635 (461‐852)

Numbers (%) or medians (IQR) are shown.

cART, combination antiretroviral therapy; CDC, Centers for Disease Control and Prevention; IQR, interquartile range.

### 3.2 Trends in suicide rates in people living with HIV and the general population

**Figure 1:**
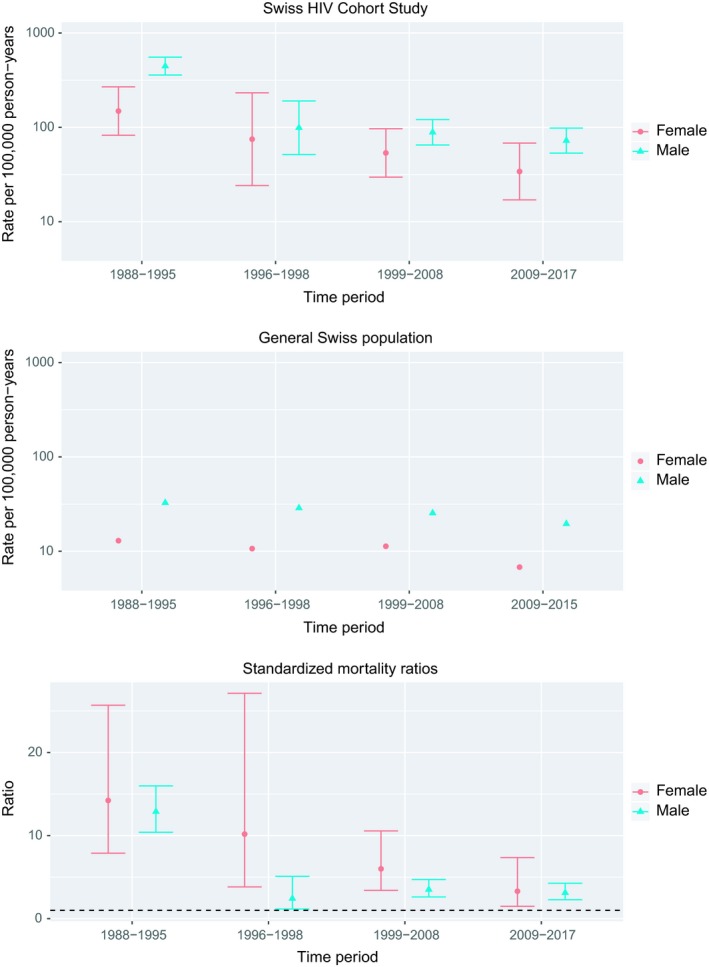
Suicide in the Swiss HIV Cohort Study and the general population, 1988‐2017, by gender. Rates in the cohort (upper panel), the general population (middle panel) and standardized mortality ratios (SMRs; bottom panel). Rates and SMRs with 95% confidence intervals are shown.

Overall, 204 people with HIV died by suicide over 184,402 person‐years, for a rate of 110.6 per 100,000 person‐years (95% CI 96.4‐126.9). Figure 1 shows trends in rates of suicide in comparison to the general Swiss population. In men living with HIV, crude suicide rates decreased from 446.1 per 100,000 person‐years (95% CI 358.8‐554.7) in the pre‐cART era, to 99.1 (95% CI 51.6‐190.5) in the two years following the introduction of cART, then to 72.4 (95% CI 55.3‐96.4) in the later cART era. In women, the corresponding decline was from 148.9 (95% CI 82.5‐268.9), to 74.9 (95% 24.2‐232.3), then to 34.0 (95% CI 17.0‐68.1). Crude rates also declined in the general Swiss population from the pre‐cART era to the later cART era: from 32.6 (95% CI 32.1‐33.2) to 19.5 (95% CI 19.0‐20.1) in men, and from 12.9 (95% CI 12.6‐13.2) to 6.8 (95% CI 6.5‐7.1) in women. The SMRs comparing men living with HIV with men from the general population decreased from 12.9 (95% CI 10.4‐16.0) in the pre‐cART era, to 2.4 (95% CI 1.2‐5.1) after the introduction of cART, after which it increased slightly to 3.1 (95% CI 2.3‐4.3) in the later cART era. In women, the corresponding ratios declined from 14.2 (95% CI 7.9‐25.7) to 10.2 (3.8‐27.1), and to 3.3 (95% CI 1.5‐7.4).

The biggest drop in rates was thus observed after cART was introduced in 1996. Since then no important decline in rates was observed in men, despite the more tolerable, efficacious and simpler antiretroviral drug regimens that became available in the later cART period 17. In contrast, rates continued to decline in women. Rates also decreased in the general population, and the suicide rates among people living with HIV continued to be over three times higher compared to the general Swiss population, both in women and men.

**Figure 2:**
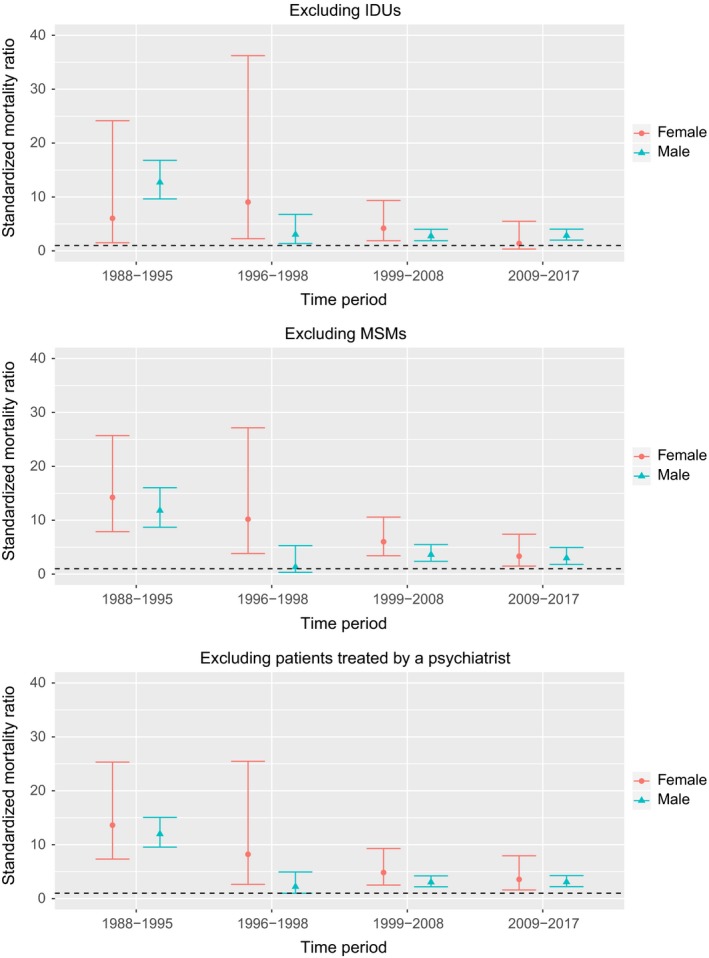
Standardized mortality ratios (SMRs) comparing suicide rates in the Swiss HIV Cohort Study and the general population, after excluding individuals who had contracted HIV through intravenous drug use (upper panel), or men who had contracted HIV through sex with other men (middle panel), or patients undergoing psychiatric treatment at enrollment (bottom panel). SMRs with 95% confidence intervals are shown. IDU, intravenous drug use; MSM, men who have sex with men.

Results were similar after excluding men who have sex with men (MSM) or patients being treated by a psychiatrist at enrollment (Figure 2). When excluding individuals who contracted HIV through IDU, results were similar in men, whereas in women the SMR was close to 1 in the later cART period, with wide confidence intervals.

### 3.3 Risk factors for suicide in people living with HIV

Participants of the SHCS who died by suicide were more likely to be male, of Swiss origin, have a history of IDU and have a lower CD4 cell count at the last visit than patients who did not die by suicide. In the two cART eras, but not in the pre‐cART era, those who died by suicide were also more likely to be in a more advanced clinical stage, and more likely to have been in psychiatric treatment (Table 1). Table 2 summarizes risk factors for suicide. All variables except age were important predictors of suicide over the entire study period. When analysed by period, psychiatric treatment was a strong predictor for suicide in all three eras, with HRs ranging from 2.41 to 3.67. Men and Swiss nationals had higher rates of suicide than women and other nationals, and patients with less advanced disease (stage A) had lower rates than those with more advanced disease, but these associations did not reach conventional levels of statistical significance for each ART era. When formally assessing trends across periods using interaction tests, there was little evidence that associations with risk factors changed (*p* values from interaction tests all > 0.1), in contrast to the previous analysis of the SHCS data up to the year 2008 10.

**Table 2:** Risk factors for suicide in the Swiss HIV Cohort Study during the pre‐cART (1988‐1995), earlier cART (1996‐2008) and later cART (2009‐2017) eras

Variable	Pre‐cART era	Earlier cART era	Later cART era	All eras	
	aHR (95% CI)	aHR (95% CI)	aHR (95% CI)	aHR (95% CI)	*p*
Age (per 10‐year increase)	1.06 (0.81‐1.38)	1.29 (0.99‐1.70)	1.17 (0.85‐1.60)	0.98 (0.84‐1.15)	0.79
Sex					0.009
Male	1	1	1	1	
Female	0.46 (0.23‐0.89)	0.84 (0.43‐1.64)	0.62 (0.26‐1.48)	0.58 (0.38‐0.87)	
Origin					0.001
Other	1	1	1	1	
Swiss	1.33 (0.75‐2.36)	2.68 (1.21‐5.94)	1.23 (0.63‐2.38)	1.95 (1.34‐2.83)	
Risk group					<0.001
Other	1	1	1	1	
Men having sex with men	2.23 (1.05‐4.74)	1.72 (0.82‐3.61)	1.82 (0.80‐4.16)	2.10 (1.36‐3.24)	
Injection drug use	2.02 (0.97‐4.23)	1.85 (0.88‐3.86)	2.65 (1.09‐6.42)	2.64 (1.71‐4.09)	
CDC clinical stage					<0.001
C	1	1	1	1	
B	0.63 (0.38‐1.03)	0.63 (0.34‐1.18)	0.35 (0.16‐0.81)	0.55 (0.39‐0.78)	
A	0.28 (0.17‐0.47)	0.57 (0.31‐1.05)	0.55 (0.28‐1.06)	0.33 (0.24‐0.46)	
Psychiatric treatment					<0.001
No	1	1	1	1	
Yes	2.45 (1.45‐4.14)	3.63 (2.14‐6.16)	2.42 (1.32‐4.43)	2.32 (1.71‐3.14)	

aHR, adjusted hazard ratio; cART, combination antiretroviral therapy; CDC, Centers for Disease Control and Prevention.

We found that living alone was associated with higher suicide rates (aHR 1.83, 95% CI 1.17‐2.87) during 2002‐2017, independently of the other factors. We found no such association with education and unemployment. Furthermore, adjusting for these socio‐economic factors did not noticeably alter the estimated effect of the other variables on suicide rates.

Among individuals on cART, those with a history of psychiatric treatment at enrollment were less likely to be prescribed efavirenz compared to those without (aOR 0.70; 95% CI 0.60‐0.81). Similarly, participants with a history of IDUs were less likely to be prescribed efavirenz than patients in the other transmission groups (aOR 0.58; 95% CI 0.51‐0.65). Women were less likely to receive efavirenz than men (aOR 0.87; 95% CI 0.79‐0.95, comparing women to men). Dolutegravir was more likely to be prescribed to Swiss nationals than to non‐Swiss (aOR 1.28; 95% CI 1.10‐1.49), and to patients with advanced CDC clinical stage. We found no evidence that efavirenz‐based therapy was associated with suicide in HIV‐positive people on cART (aHR 1.00, 95% CI 0.66‐1.52). The analysis for dolutegravir‐based cART was similarly inconclusive (aHR 0.38, 95% CI 0.09‐1.57).

Male gender, Swiss nationality, a history of injection drug use, sexual contact between men, or of treatment by a psychiatrist and being at a more advanced clinical stage were thus risk factors for suicide among HIV‐positive people living in Switzerland, confirming risk factors found in earlier studies from France and Denmark 9, 18. Of note, there was little evidence that the strength of the associations with these risk factors changed over the study period, in contrast to a previous analysis of the SHCS including data up to the year 2008 10. The lack of an association with efavirenz supports the notion that in clinical practice, efavirenz may be less frequently used in patients with underlying psychiatric conditions 19.

### 3.4 Strengths and limitations

Strengths of this study include the large number of patients followed up during almost 30 years. The SHCS enrolled about 45% of all patients with HIV and about 70% of patients with AIDS in the country 15, and results are likely to be applicable to Switzerland and similar countries. Another strength was the inclusion of data from the SNC 16, which allowed us to compare time trends in suicide rates and risk factors between HIV‐infected patients and the general population.

Our study has several limitations. We standardized the SMRs with respect to age, calendar year, and gender, but not with respect to sexual orientation, IDU, or other lifestyle factors that could affect suicide rates. Another limitation of our study is the possible misclassification of suicide and death by accidental overdose in participants with a history of IDU. Suicide attempts were not collected. Furthermore, we could not distinguish between suicide and assisted suicide. The latter is legal in Switzerland under certain conditions 20, but is not specifically reported in the SHCS. Assisted suicide rates have increased in Switzerland over the past decade, but this increase mainly concerned the elderly 21, 22.

### 3.5 Interpretation and context

Sexual orientation, substance use and other mental health problems may be distal common causes of HIV infection and suicide, with HIV‐related stigma, addiction, and depression on the proximal causal pathway to suicide 23. These variables were not available for the general population in this study, precluding, for example, comparisons of suicide rates in MSM with heterosexual men. Meta‐analysis of cross‐sectional studies of lifetime suicide attempts showed that the risk was higher in lesbian, gay and bisexual people than heterosexual people, and particularly high in gay and bisexual men 24. A Danish study 25, but not a study from the United States of America 26, showed that mortality from suicide was higher in MSM than in heterosexual men. Our sensitivity analyses excluding people who acquired HIV through IDU or through MSM are compatible with the hypothesis that the increased risk of suicide in HIV‐positive women may mainly be driven by IDU, whereas in men it may be related to both to IDU and the higher incidence in MSM, mediated by stigma, isolation and discrimination. In a French survey, poor socio‐economic status was shown to be associated with an increased risk of suicide attempts in women with HIV 27, while loneliness and discrimination were also associated with a higher risk of suicide, independently of sex and sexual orientation 28.

How do suicide rates in people with HIV compare with those suffering from other conditions? Suicide rates have been shown to be higher in people with conditions associated with chronic pain or poor prognosis 29, including patients with cancer 30, neurological diseases such as amyotrophic lateral sclerosis 31 or progressive multiple sclerosis 32. An analysis of the Surveillance, Epidemiology and End Results database 1973 to 2013 in the United States of America showed that SMRs for suicide ranged from 1.18 for prostate cancer to 4.17 for lung cancer 33. SMRs decreased over time for all cancers, but particularly for lung cancer. An increased risk of suicide has also been shown for several neurological disorders, including multiple sclerosis, stroke, Huntington disease and epilepsy, whereas Parkinson's disease may be associated with a reduced risk of suicide 34. Of note, in a nationwide sample of people with HIV on cART in the Netherlands, poor mental health was more common in people living with HIV compared to people living with diabetes or rheumatoid arthritis 35.

## 4 Conclusions

Since the introduction of cART in 1996, mortality rates due to suicide have significantly decreased in men and women living with HIV in Switzerland, but no important improvements in SMRs were observed in men in more recent years, despite the advent of more tolerable, more efficacious and less complex antiretroviral drug regimens in the later cART period 17. Furthermore, it is noteworthy that the SMRs were larger than those reported in the literature for conditions with worse prognosis. In Switzerland and elsewhere, there is a continued need to monitor depression, suicidality in HIV‐positive people, and to develop tailored suicide prevention programs aimed at reducing suicide risk in people living with HIV.

## Competing interests

The authors declare no conflict of interest. HFG has received unrestricted research grants from Gilead Sciences and Roche; fees for data and safety monitoring board membership from Merck; consulting/advisory board membership fees from Gilead Sciences, Sandoz and Mepha.

## Authors’ contributions

ME, LL and OK wrote the study protocol. YR performed the statistical analyses and wrote the first draft of the paper, which was revised by ME, LL and OK. All authors contributed to collecting data, commented on the manuscript and approved the final version.

## Appendix 1

### 1.1

The members of the Swiss HIV Cohort Study (SHCS) are Anagnostopoulos A, Battegay M, Bernasconi E, Böni J, Braun DL, Bucher HC, Calmy A, Cavassini M, Ciuffi A, Dollenmaier G, Egger M, Elzi L, Fehr J, Fellay J, Furrer H, Fux CA, Günthard HF (President of the SHCS), Haerry D (deputy of ‘Positive Council’), Hasse B, Hirsch HH, Hoffmann M, Hösli I, Huber M, Kahlert CR (Chairman of the Mother & Child Substudy), Kaiser L, Keiser O, Klimkait T, Kouyos RD, Kovari H, Ledergerber B, Martinetti G, Martinez de Tejada B, Marzolini C, Metzner KJ, Müller N, Nicca D, Paioni P, Pantaleo G, Perreau M, Rauch A (Chairman of the Scientific Board), Rudin C, Scherrer AU (Head of Data Centre), Schmid P, Speck R, Stöckle M (Chairman of the Clinical and Laboratory Committee), Tarr P, Trkola A, Vernazza P, Wandeler G, Weber R, Yerly S TBD.
